# Retroperitoneal dermoid cyst in a young boy

**DOI:** 10.11604/pamj.2021.38.36.27680

**Published:** 2021-01-14

**Authors:** Saket Kumar, Venkat Rao Chidipotu

**Affiliations:** 1 Department of Surgical Gastroenterology, Indira Gandhi Institute of Medical Sciences, Patna, Bihar, India

**Keywords:** Retroperitoneal, dermoid cyst, teratoma

## Image in medicine

A 12-year-old boy presented with history of pain in the right flank for last the 6 months. He had no associated urinary or bowel complains. Abdominal palpation revealed a non-tender lump was felt in the right lumbar region. Lump was firm with a well-defined margin and was not moving with the respiration. The blood investigations were within normal limits. An abdominal CT scan was obtained that showed a well-defined, heterogeneous mass in right suprarenal region measuring 11.5 x 11 cm. The lesion contained fat and soft tissue attenuation along with areas of calcification. The right kidney was displaced inferiorly and the right adrenal gland was not seen separately from the lesion. Based on the physical examination and radiological findings a provisional diagnosis of retroperitoneal dermiod cyst was made. Surgical resection of the retroperitoneal dermoid cyst was performed. The lesion was placed between the right hepatic lobe and the right kidney; however, the right adrenal gland could not be defined during the operation. The tumor was composed of mature tissues such as hair, bones, and teeth along with turbid yellowish fluid and pultaceous material. Histopathology was consistent with the diagnosis of a mature cystic teratoma without malignant components. Patient recovered uneventfully and was discharged on post-operative day 5.

**Figure 1:**
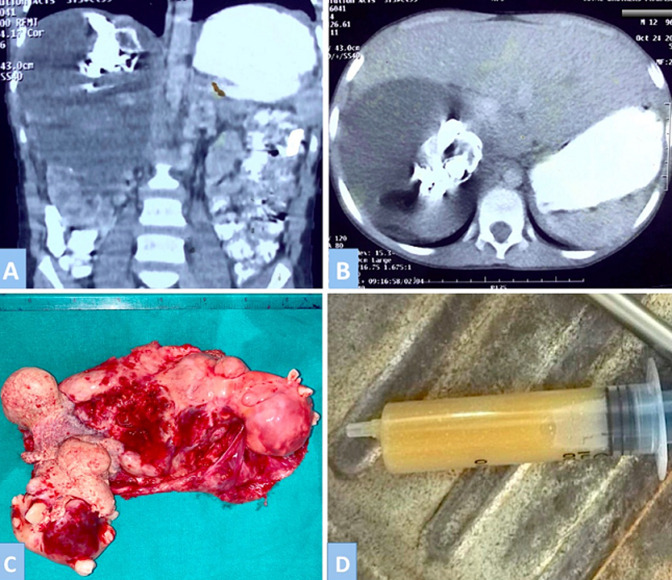
the CT scan: A) coronal; B) axial view showing a large solid-cystic lesion in the right hepatorenal fossa with areas of calcifications and foci of fat; C) resected specimen showing mature tissues such as bone, teeth and hair; D) turbid yellowish fluid aspirated from the cyst

